# Inhibition of Late and Early Phases of Cancer Metastasis by the NF-κB Inhibitor DHMEQ Derived from Microbial Bioactive Metabolite Epoxyquinomicin: A Review

**DOI:** 10.3390/ijms19030729

**Published:** 2018-03-03

**Authors:** Yinzhi Lin, Tamami Ukaji, Naoki Koide, Kazuo Umezawa

**Affiliations:** 1 Department of Molecular Target Medicine, Aichi Medical University School of Medicine, 1-1 Yazako-Karimata, Nagakute 480-1195, Japan; lin.yinzhi.026@mail.aichi-med-u.ac.jp; 2 Department of Microbiology and Immunology, Aichi Medical University School of Medicine, 1-1 Yazako-Karimata, Nagakute 480-1195, Japan; ukaji@aichi-med-u.ac.jp (T.U.); koide@aichi-med-u.ac.jp (N.K.)

**Keywords:** epoxyqinomicin, DHMEQ, metastasis, invasion, adhesion, 3D cell culture

## Abstract

We previously designed and synthesized dehydroxyepoxyquinomicin (DHMEQ) as an inhibitor of NF-κB based on the structure of microbial secondary metabolite epoxyquinomicin C. DHMEQ showed anti-inflammatory and anticancer activity in various in vivo disease models without toxicity. On the other hand, the process of cancer metastasis consists of cell detachment from the primary tumor, invasion, transportation by blood or lymphatic vessels, invasion, attachment, and formation of secondary tumor. Cell detachment from the primary tumor and subsequent invasion are considered to be early phases of metastasis, while tumor cell attachment to the tissue and secondary tumor formation the late phases. The assay system for the latter phase was set up with intra-portal-vein injection of pancreatic cancer cells. Intraperitoneal administration of DHMEQ was found to inhibit liver metastasis possibly by decreasing the expression of MMP-9 and IL-8. Also, when the pancreatic cancer cells treated with DHMEQ were inoculated into the peritoneal cavity of mice, the metastatic foci formation was inhibited. These results indicate that DHMEQ is likely to inhibit the late phase of metastasis. Meanwhile, we have recently employed three-dimensional (3D) culture of breast cancer cells for the model of early phase metastasis, since the 3D invasion just includes cell detachment and invasion into the matrix. DHMEQ inhibited the 3D invasion of breast cancer cells at 3D-nontoxic concentrations. In this way, DHMEQ was shown to inhibit the late and early phases of metastasis. Thus, DHMEQ is likely to be useful for the suppression of cancer metastasis.

## 1. Introduction

Microbial and plant-derived bioactive metabolites are valuable sources of organic compounds having various structures and biological activities. On the other hand, many diseases can be explained by abnormality of specific cellular signaling at present. We have isolated various signal transduction inhibitors of low molecular weight from microbial and plant secondary metabolites. These inhibitors include protein-tyrosine kinase inhibitor lavendustin from *Streptomyces* [1], protein-tyrosine phosphatase inhibitor dephostatin from *Streptomyces* [2], anti-K-Ras compound aglaiastatin from a plant [3], phosphatidylinositol-specific phospholipase C inhibitor akaterpin from a marine sponge [4], lipopolysaccharide inactivating compound heptadepsin from a bacterium [5], and inhibitor of cancer cell migration migracin from *Streptomyces* [6]. These are all novel compounds. We have also found that a plant-derived alkaloid conophylline induces pancreatic β-cell differentiation and protects islets from fibrosis in animals [7]. Thus, these bioactive metabolites should be useful to study the mechanism of diseases, and they can be developed as new therapeutic agents. More recently, we discovered NF-κB inhibitor dehydroxymethyl-epoxyquinomicin (DHMEQ) by molecular design based on the structure of naturally occurring epoxyquinomicin C. In the present review, we describe inhibition of early and late phases of cancer metastasis by DHMEQ.

## 2. Discovery and Mechanism of Action of NF-κB Inhibitor DHMEQ

**Figure 1:**
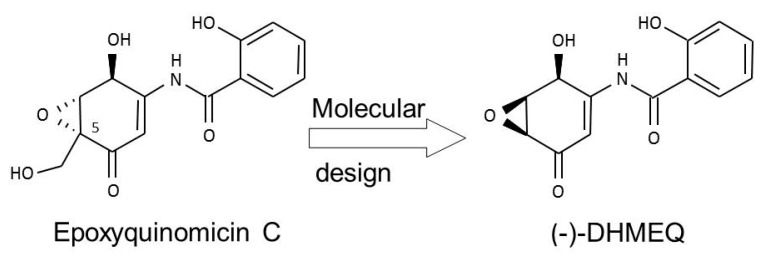
Molecular design of (−)-DHMEQ as an inhibitor of NF-κB based on the structure of antibiotic epoxyquinomicin C.

In the course of our search for chemical inhibitors of NF-κB, we designed and synthesized new NF-κB inhibitors with reference to the structure of epoxyquinomicin C (Figure 1) [8]. Epoxyquinomicin C is a weak and useless antibiotic, but shows no toxicity in animals. Although related compounds such as panepoxydone [9] and cycloepoxydone [10] inhibit NF-κB, epoxyquinomicin C do not inhibit NF-κB. However, after the removal of the protruding hydroxymethyl moiety, the designed compound dehydroxymethylepoxyquinomicin (DHMEQ, Figure 1), did inhibit NF-κB activity. After synthesis, we found that DHMEQ ameliorated inflammation in a collagen-induced rheumatoid arthritis in mice when administered by the intraperitoneal (IP) route [8]. Thus, we found a new NF-κB inhibitor active in vivo.

**Figure 2:**
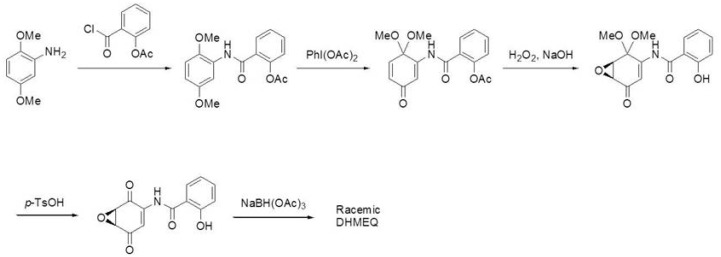
Synthesis of racemic DHMEQ. Racemic DHMEQ is synthesized from 2,5-dihydroxyaniline in five steps.

Racemic DHMEQ can be synthesized from a 2,5-dimethoxyaniline in five steps [11], and can be separated into each enantiomer practically by lipase [12]. Lipase reacts with racemic dihexanoyl-DHMEQ to give (−)-DHMEQ and monohexanoyl-(+)-DHMEQ that can be easily removed by difference of solubility. (−)-DHMEQ is about ten times more effective than (+)-DHMEQ in inhibiting NF-κB [11]. (−)-DHMEQ is mainly used for the cellular experiments, and racemic DHMEQ for the animal experiments. Synthetic pathway for DHMEQ is shown in Figure 2 [11].

**Figure 3:**
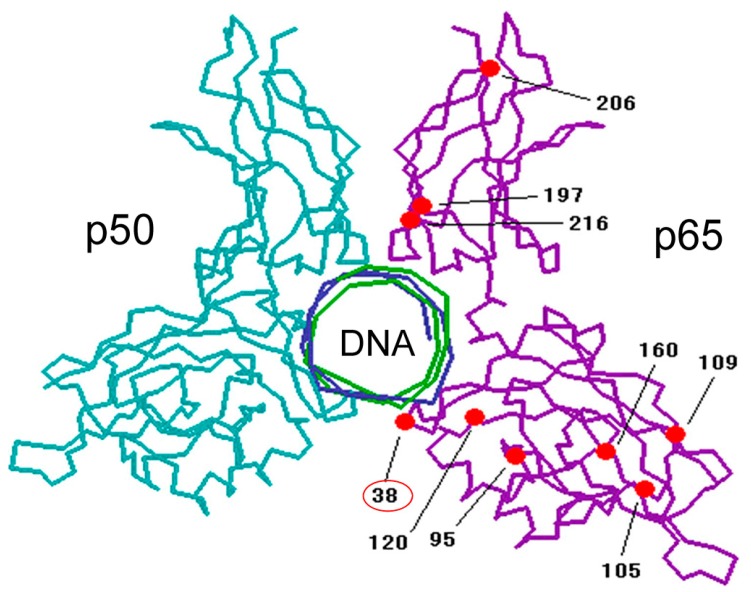
(−)-DHMEQ binds to Cys38 in p65. Cysteine residues are shown in red.

For the mechanism of DHMEQ, we firstly reported that it inhibited the nuclear translocation of NF-κB [13]. However, more recently, we found that DHMEQ directly binds to the Rel-family proteins to inhibit their DNA-binding activity [14]. Inhibition of NF-κB nuclear translocation is likely to be a result after the inhibition of DNA binding [15]. Rel family proteins are the constituents of NF-κB molecules including p65, Rel B, c-Rel, p50, and p52. (−)-DHMEQ binds to p65 covalently with a 1:1 stoichiometry as revealed by surface plasmon resonance (SPR) and MALDI-TOF mass spectrum (MS) analyses. MS analysis of the chymotrypsin-digested peptide suggested the binding of (−)-DHMEQ to a specific cysteine residue. In the case of p65, DHMEQ only binds to the Cys38 residue, which is located close to the DNA (Figure 3). Observation of the adduct in MALDI-TOF MS suggests that the (−)-DHMEQ-cysteine binding is a covalent one. The formation of (−)-DHMEQ-cysteine covalent binding in the protein was supported by chemical synthesis of the conjugate molecule [16]. Since (−)-DHMEQ binds to the cysteine residue covalently in an NF-κB molecule, the inhibitory effect is irreversible. LPS induces NF-κB activation in 30 min in a macrophage-like mouse cell line RAW264.7. (−)-DHMEQ was added for only 15 min and then washed out in the RAW264.7 cell experiment. After 8 h of the removal of (−)-DHMEQ, LPS could not activate NF-κB, suggesting NF-κB was inhibited irreversibly [17].

All Rel family proteins possess specific cysteine residues essential for their DNA binding. (−)-DHMEQ binds to p65, cRel, RelB, and p50, but not to p52 at specific cysteine residues. (−)-DHMEQ is the first NF-κB inhibitor shown to covalently bind to a specific cysteine. We have also found that (−)-DHMEQ inhibits not only DNA-binding of RelB, but also its interaction to importin [18]. It also induces instability of RelB.

Thus, (−)-DHMEQ specifically binds to a cysteine residue in both the canonical and the noncanonical NF-κB components [14,18]. It is likely that DHMEQ can enter into a specific pocket via a key and lock mechanism to bind limitedly to the limited cysteine residue. These findings may explain the highly selective NF-κB inhibition and the low toxic effect of DHMEQ in cells and in animals.

## 3. Therapeutic Activity of DHMEQ on Inflammatory and Neoplastic Disease Models

DHMEQ was used for many animal experiments to suppress various inflammation and neoplastic diseases and to study the mechanism of diseases. Recent results on its anti-inflammatory and anticancer activities are described below.

DHMEQ is now being developed as an ointment drug for the treatment of atopic dermatitis and other severe skin inflammations. In the genetic atopic dermatitis model, DHMEQ ointment showed similar or stronger anti-inflammatory activities compared with betamethasone and tacrolimus ointments [19]. Accumulation of mast cells was inhibited by DHMEQ ointment in this model. Later DHMEQ was found to inhibit MMP-2 expression and cellular invasion of mouse primary culture mast cells treated with DNP antigen and IgE [20]. More recently, DHMEQ ointment was shown to suppress development of chemically induced atopic dermatitis-like lesions in BALB/c mice [21]. Atopic dermatitis-like lesions were chronically induced by the repetitive and alternative application of 2, 4-dinitrochlorobenzene (DNCB) and oxazolone (OX) on ears. The mice were then externally treated with DHMEQ ointment. DHMEQ inhibited ear swelling and relieved clinical symptoms of the atopic dermatitis-like lesions induced by DNCB/OX in BALB/c mice. Histopathology examination illustrated that it significantly decreased DNCB/OX-induced epidermal thickness, the infiltration of inflammatory cells, and the count of mast cells. The elevated level of IgE in serum and the mRNA levels of IL-4 and IL-13 in the ear tissues were also suppressed by DHMEQ.

Amniotic apoptosis is essential for the onset of delivery. On the other hand, too early amniotic apoptosis causes baby loss. Amniotic apoptosis is induced by the TNF-α pathway via the TNF-α receptor 1 expressed in the amniotic epithelial cells. Activated macrophages may cause amniotic apoptosis mediated by TNF-α and NO expression and production. DHMEQ inhibited TNF-α and iNOS expressions in pregnant mice to inhibit amniotic apoptosis [22].

DHMEQ ameliorates organ transplantation in many ways acting as an immunosuppressant. Recently, DHMEQ was found to ameliorate graft-versus-host disease (GVHD) in allogeneic bone marrow transplantation [23]. GVHD is a crucial mortality factor in allogeneic bone marrow transplantation. DHMEQ suppressed GVHD, resulting in an improved mortality rate in a mouse allogeneic bone marrow transplantation model. Bone marrow cells from C57BL/6 mice (B6 mice) were transplanted into lethally irradiated BALB/c mice. Two weeks later, spleen cells from B6 mice were transplanted into the irradiated BALB/c mice. From one week after the injection of spleen cells, when the mice started to show GVHD, the mice were also IP-injected daily with DHMEQ or vehicle for four weeks. By 80 days after the transplantation, 6/14 of the vehicle-injected mice (43%) had died because of GVHD, whereas all DHMEQ-injected mice survived this observation period and developed milder GVHD than the vehicle-injected mice. These findings suggest that administration of DHMEQ would become a new strategy for preventing fatalities from GVHD.

Recently, it was reported that IP administration of DHMEQ ameliorates dinitrobenzene sulfonic acid (DNBA)-induced colitis in rats [24]. IP administration of DHMEQ also inhibited dextran-sulfate-sodium-induced colitis in rats [25].

NF-κB in cancer cells often induces immunosuppression in cancer patients to facilitate the cancer progression. DHMEQ inhibited the NF-κB-dependent immunosuppression in ovarian carcinoma in animal experiment [26]. Although T-cell immunity is considered to be involved in the prognosis of epithelial ovarian cancer patients, immunosuppressive conditions often occurs to hamper antitumor immune responses. In epithelial ovarian cancer patients, increase of plasma IL-6, and IL-8 were observed. DHMEQ was found to inhibit the production of IL-6 and IL-8 in epithelial ovarian cancer cell lines. IP administration of DHMEQ to nude mice implanted with human epithelial ovarian cancer cells resulted in the restoration of T-cell stimulatory activity of murine dendritic cells along with the reduction of tumor accumulation. Inhibition of NF-κB in tumor-bearing mice also enhanced antitumor effects of transferred murine naive T cells. Thus, NF-κB is involved in the immunosuppression induced by human epithelial ovarian cancer cells, and its inhibitor such as DHMEQ may restore anticancer immune responses.

DHMEQ inhibits cancer progression in various animal models. Cholangiocarcinoma is one of the most difficult cancers to treat, and there is no effective chemotherapeutic regimen at present. Seubwai and coworkers reported that IP administration of DHMEQ inhibited the growth of cholangiocarcinoma in mice [27]. Normal bile duct epithelia rarely expressed NF-κB subunits such as p50, p52 and p65, whereas all cholangiocarcinoma patient tissues over-expressed these NF-κB subunits. DHMEQ increased cell apoptosis by decreasing the expressions of anti-apoptotic proteins such as Bcl-2 and XIAP. Moreover, DHMEQ effectively reduced tumor size in cholangiocarcinoma-inoculated mice.

Ito and coworkers investigated the anticancer effect of DHMEQ in CDDP-resistant bladder cancer cells [28]. Invasive bladder carcinoma cell line T24 and its CDDP-resistant cell line T24PR were used. The NF-κB activity was stronger in T24PR cells than in T24 cells. Lowered cell viability and strong induction of apoptosis were observed by treatment with DHMEQ alone in T24PR cells compared with T24 cells. T24PR cells did not show dramatic cross-resistance to paclitaxel in the in vitro study. They next examined whether the combination of DHMEQ with paclitaxel could enhance the therapeutic effect of the paclitaxel treatment in T24PR tumors. Using mouse xenograft models, the mean volume of tumors treated with the combination of DHMEQ and paclitaxel was significantly smaller than those treated with paclitaxel alone. Thus, DHMEQ showed anticancer activity alone, and also increased the sensitivity to paclitaxel.

**Figure 4:**
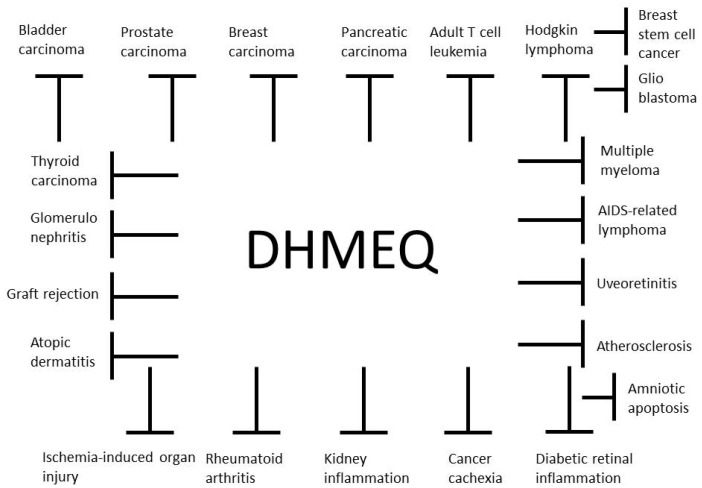
Anti-inflammatory and anticancer activities of DHMEQ in animal models.

Anti-inflammatory and anticancer activities of DHMEQ in animal models are summarized in Figure 4. DHMEQ was given to animals by IP injection in most cases. The mechanism of effect by IP administration is discussed below. It was given by ointment for the atopic dermatitis model [19] and by subcutaneous injection for the AIDS-related lymphoma model [29]. With ointment or SC administration, DHMEQ is considered to reach the inflammatory or cancer site directly.

DHMEQ is given to the animals at 2–12 mg/kg every day or three times a week by IP injection. There has been no toxicity observed so far. DHMEQ was given to mice three times a week at 4 mg/kg for more than six months to suppress adult T-cell leukemia, and no toxicity was observed in this experiment [30]. IP administration of DHMEQ at 20 mg/kg every day for 14 days did not decrease whole blood cell count, hemoglobin content, and platelet count in mice (unpublished results). Thus, DHMEQ is a comparatively non-toxic compound as shown by the results of many animal experiments.

## 4. Contribution of NF-κB to Cancer Metastasis

Meanwhile cancer cell proliferation and metastasis are often influenced by the microenvironment. The microenvironment consists of various cell types including endothelial cells forming lymph and blood vessels, fibroblasts, and bone marrow derived cells such as macrophages, neutrophils, and mast cells. The tumor microenvironment contributes to tumor progression by secretion of growth factors, cytokines and chemokines. These cytokine and chemokine transcriptions are often dependent on NF-κB. Besides providing growth factors and cytokines, the tumor microenvironment promotes tumor progression by extracellular matrix (ECM) degradation mainly by matrix metallo-proteases (MMPs). Up-regulated MMP secretion is found in many cancer cells. MMP secretions were shown to associate with enhanced cell proliferation, migration, angiogenesis, metastasis and poor survival. The functions of MMPs are (i) cleaving cell adhesion molecules such as E-cadherin; (ii) the degradation of ECM proteins; and (iii) the processing and activation of cytokines and growth factors. Even natural immune cells such as macrophages of the tumor microenvironment often execute a tumor-promoting rather than a tumor-suppressing role. It is well established that chronic infections and inflammation frequently lead to cancer development and enhancement of tumor progression. Interestingly, NF-κB has been identified as a key activating signaling pathway in both cancer cells and tumor-associated immune cells. Expression of cytokines such as IL-1, IL-6, TNF-α and many MMPs such as MMP-9, MMP-13, and MT1-MMP is dependent on NF-κB. These protein expressions and productions cause inflammation in the tissue, and cancer cell proliferation and metastasis.

## 5. Inhibition of Late and Early Phases of Metastasis by DHMEQ

**Figure 5:**
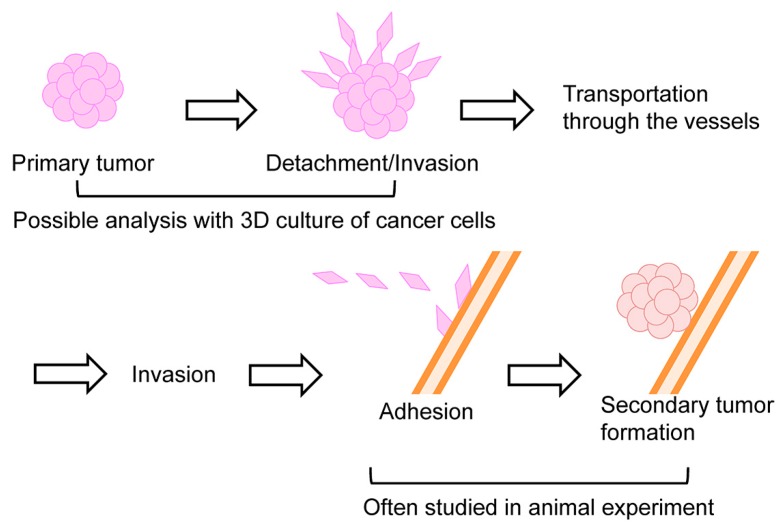
Several steps of metastasis. The early phase including cell detachment and invasion can be studied by 3D invasion of cultured cells, and later phase including invasion and attachment is commonly studied by injection of cancer cells into the transporting veins in animal experiments.

**Figure 6:**
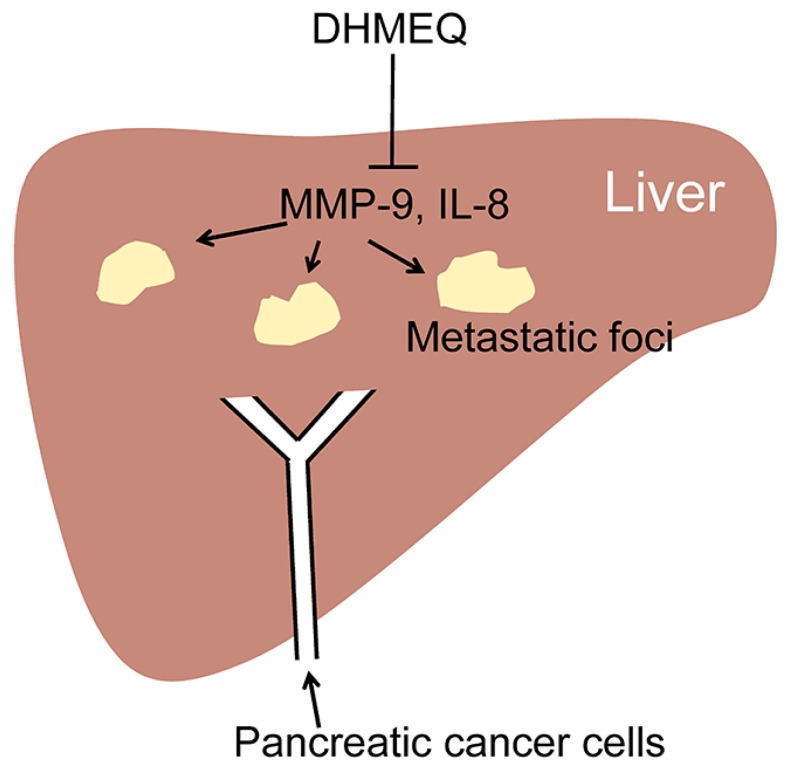
Inhibition of pancreatic cancer cell metastasis in liver by intraperitoneal administration of DHMEQ in mice.

The process of cancer metastasis consists of cell detachment from the primary tumor, invasion, transportation by blood or lymphatic vessels, invasion, attachment, and formation of secondary tumor (Figure 5). Typical in vivo metastasis model is lung metastasis of human cancer cells in rodents. In this model, the cancer cells are inoculated into the tail vein. We previously found that lung metastasis of Yoshida ascites hepatoma AH7974 cells was inhibited by intraperitoneal administration of protease inhibitor leupeptin [31]. On the other hand, tumors in pancreas often metastasize into the liver. Suzuki and coworkers investigated the effects of DHMEQ on the inhibition of liver metastasis of human pancreatic cancer in a mouse model [32]. Nude mice were xenografted by intra-portal-vein injection with human pancreatic adenocarcinoma AsPC-1 cells via laparotomy (Figure 6). Mice were treated with DHMEQ and gemcitabine, alone or in combination. DHMEQ alone inhibited the metastasis. The combination of gemcitabine and DHMEQ showed a stronger antitumor effect than either monotherapy. Apoptosis induction in the metastatic foci was most prominent in the DHMEQ and gemcitabine group. Significant reductions in the numbers of neovessels were also seen in the DHMEQ and/or gemcitabine groups. On the other hand, cell growth inhibition assays revealed no synergistic effect of combination therapy, although each monotherapy had an individual cytotoxic effect. For the mechanism, DHMEQ alone markedly down-regulated expressions of MMP-9 and interleukin (IL)-8 in metastatic foci. These results demonstrate that DHMEQ can exert anticancer effects by inhibiting angiogenesis and tumor cell invasion. Combination therapy with DHMEQ and gemcitabine also showed potential efficacy. Thus, DHMEQ may be useful for the treatment of advanced pancreatic cancer.

NF-κB is also involved in resistance to anoikis, a special type of apoptosis induced when cells are detached from the extracellular matrix or other cells. Anoikis resistance is related to the metastatic abilities of tumor cells. Sato and coworkers employed DHMEQ to investigate anoikis induction and peritoneal metastasis suppression following the cellular NF-κB inhibition [33]. Pancreatic cancer AsPC-1 cells were photo-marked by Gluc, a secretory form of luciferase for in vivo experiments. DHMEQ induced anoikis in AsPC-1-Gluc cells as measured by the cell survival assays and flow cytometry. DHMEQ inhibited cellular NF-κB and the subsequent expressions of anti-apoptotic molecules. In a murine xenograft model, anoikis-resistant PC cell lines tended to metastasize to the peritoneum more than anoikis-sensitive cells, suggesting a correlation between anoikis sensitivity and peritoneal metastasis. DHMEQ inhibited peritoneal metastasis of AsPC-1-Gluc cells. They monitored metastasis inhibition by ex vivo chemiluminescent detection of Gluc secreted from tumor cells into murine plasma and by in vivo imaging. Thus, DHMEQ inhibited peritoneal dissemination by preventing anoikis resistance of PC cells. These results suggest that DHMEQ can inhibit the late phase of metastasis.

**Figure 7:**
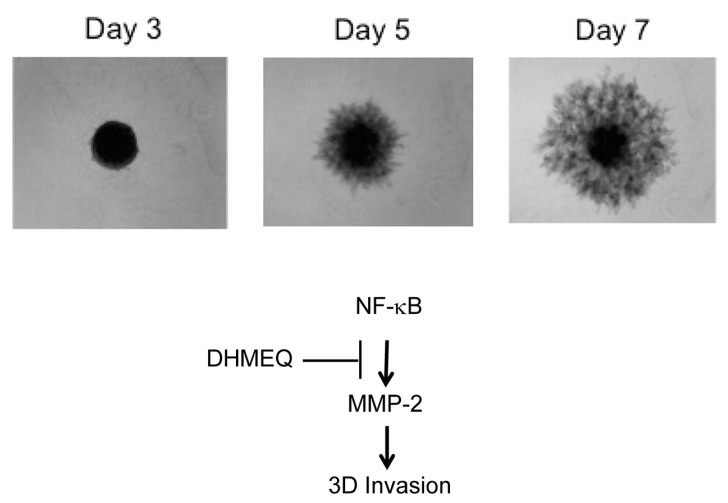
Time-course of 3D invasion in breast carcinoma MDA-MB-231 cells. (Modified from the figure in Reference 34). The cells were cultured in a 96-well round bottom well. DHMEQ inhibited 3D invasion by the decrease of MMP-2 expression.

The three-dimensional (3D) culture of cancer cells provides an environmental condition closely related to the condition in vivo. Recently, 3D culture of cancer cells has been developed with round bottom well. The cancer cells grow in soft agar forming spheroid colonies. We employed human breast carcinoma MDA-MB-231 cells for 3D culture. Then, we have found that 3D invasion of MDA-MB-231 cells from the colony is similar to the illustration of the early phase of metastasis shown in (Figure 7) [34]. We thought it would be an ideal model for the early phase of metastasis, including the detachment and invasion of cancer cells from the primary tumor. Then, we studied the inhibitory activity of DHMEQ on the 3D invasion of breast carcinoma cells. MDA-MB-231 cells showed the most active invasion from spheroid among the cell lines tested. DHMEQ inhibited the 3D invasion of cells at the 3D-nontoxic concentrations. The PCR array analysis using RNA isolated from the 3D on-top cultured cells indicated that MMP-2 expression is lowered by DHMEQ. Knockdown of MMP-2 inhibited the invasion. DHMEQ was shown to inhibit the promoter activity of MMP-2 in the reporter assay. Thus, DHMEQ was shown to inhibit NF-κB/MMP-2-dependent cellular invasion in 3D-cultured MDA-MB-231 cells, suggesting that DHMEQ would inhibit the early phase of metastasis.

## 6. Suppression of Peritoneal NF-κB May Inhibit Peripheral Inflammation and Tumor Formation

To inhibit the late phase of metastasis in animal experiments, DHMEQ was administered by IP injection [32]. IP administration of drugs is commonly carried out in in vivo experiments with rodents. It is because it is technically easily compared to intravenous (IV) administration through the tail vein. Chemicals in the peritoneal cavity often go into the circulation. Many animal experiments with DHMEQ were also carried out by IP administration. For the rheumatoid arthritis model, both IP [8] and subcutaneous (SC) [35] administration routes were effective. Mouse model of adult T-cell leukemia was also inhibited by either IP [30] or SC [36] administration. DHMEQ ointment showed a therapeutic effect on atopic dermatitis model in mice as above [19]. The SC or ointment administration would provide DHMEQ directly to the inflammatory or tumor sites as discussed above.

In the case of IP administration, the direct effect of DHMEQ at the inflammation or tumor sites may not be likely. Since DHMEQ has various anti-inflammatory and anticancer activities without any prominent toxicity, it is now being developed as an anti-inflammatory and anticancer agent. In the course of our drug development process, we came across an interesting observation. If a chemical drug achieves an adequate blood concentration, it is much easier to develop the drug. However, we could not detect a high concentration of DHMEQ in the blood after an IP injection [37,38]. IP administration of DHMEQ to rats provided a high level in the peritoneal cavity after 5 min, although the level quickly decreased by 30 min. However, almost no DHMEQ was found in the blood after the IP administration. Employing high-performance liquid chromatography (HPLC) with mass spectroscopy (MS) to detect DHMEQ in the blood, DHMEQ at 100 ng/mL was detected after the IP injection [39]. As shown above, DHMEQ is likely to be quickly absorbed by the cells in the peritoneal cavity including macrophages and other inflammatory cells. Also, short exposure of DHMEQ should be effective for the inhibition of NF-κB. In fact, as shown before, short exposure to DHMEQ did inhibit the NF-κB activation after washing [17]. DHMEQ does not accumulate in the blood, possibly because of the quick uptake and inactivation of DHMEQ by the blood cells, especially by red blood cells. Furthermore, it has been difficult to find the active metabolite derived from DHMEQ in vitro or in the body. If salicylate is formed from DHMEQ, it should be too weak to explain the in vivo anti-inflammatory activity of DHMEQ [40]. In addition, if any metabolite of DHMEQ acts as an active principle in the blood, it would also cause toxicity in animals.

Taken together, DHMEQ would be difficult to enter the systemic circulation after IP administration. Instead, it is more likely that DHMEQ acts solely in the peritoneal cavity. This hypothesis can explain all the following curious observations on the effect of DHMEQ: In cultured cells, DHMEQ is active at 3–10 µM, which is not very low. On the other hand, in animal experiments, IP administration of only 2–12 mg/kg, which is comparatively low, can suppress inflammation and cancer. Moreover, NF-κB inhibitors are considered to be toxic in general in animals, since their effect is broad and they would block functions essential for the immune system and tissue stability. Especially, NF-κB inhibitors should show bone marrow toxicity, since the expression of many hemopoietic growth factors such as GM-CSF [41] and M-CSF (CSF-1) [42] is dependent on NF-κB. However, IP administration of DHMEQ has not shown any bone marrow toxicity so far, consistent with its poor entry to the systemic circulation. Therefore, it is likely that various subcutaneous solid tumors [43], retinal inflammation [44], and brain tumors [45], all distant from the peritoneal cavity, would be suppressed by the activity peritoneal cells. So the question arises as to how the peritoneal cells can control peripheral inflammation or cancer growth. Inflammatory cytokines such IL-6 in the systemic circulation may enhance inflammation and tumor growth in the body. We demonstrated that IP administration of DHMEQ lowers the IL-6 level in the blood in a cancer cachexia model [46]. Also, DHMEQ strongly inhibits macrophage activities in cultured cells, blocking the secretion of inflammatory cytokines [47].

**Figure 8:**
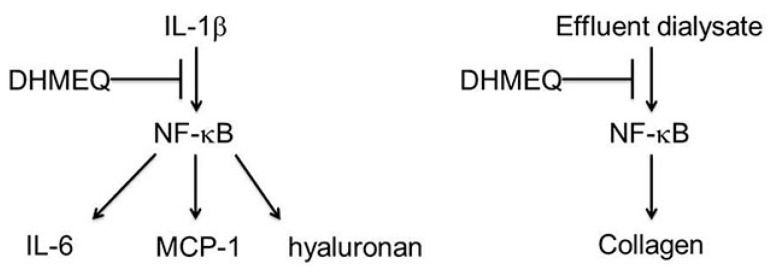
DHMEQ inhibits IL-6, MCP-1, and hyaluronan expression in human peritoneal mesothelial cells (HPMC). It also inhibits collagen production in HPMC stimulated by effluent dialysate form continuous ambulatory peritoneal dialysis.

Recently, Breborowicz and coworkers demonstrated that DHMEQ inhibited primary cultured human peritoneal cells [48]. Peritoneal mesothelial cells exposed to bioincompatible dialysis fluids often cause damage of the peritoneum during chronic dialysis. Activation of inflammatory cells in the peritoneal cavity leading to neovascularization and fibrosis plays an important role in that process. They studied the effects of DHMEQ on the function of human peritoneal mesothelial cells (HPMC) in in vitro culture. DHMEQ was not toxic at 1–10 µg/mL to HPMC. Synthesis of IL-6, MCP-1 and hyaluronan in unstimulated and stimulated with interleukin-1 was measured. DHMEQ (10 µg/mL) reduced (in unstimulated and stimulated HPMC) the synthesis of IL-6, MCP-1 and hyaluronan (Figure 8). The observed effects were due to the suppression of the expression of genes responsible for the synthesis of these molecules. DHMEQ modified the effects of the effluent dialysates from continuous ambulatory peritoneal dialysis (CAPD) patients on the function of HMPC. Dialysate induced accelerated growth of these cells, and synthesis of collagen was inhibited in the presence of DHMEQ (Figure 6). The results show that DHMEQ effectively reduces inflammatory response in HMPC and prevents dialysate-induced proliferation and collagen synthesis in these cells. These effects of DHMEQ may be beneficial during chronic peritoneal dialysis and prevents progressive dialysis-induced damage to the peritoneum.

In 1969, Vernon-Roberts reported that peritoneal cavity is an important place for macrophage differentiation in the peritoneal cavity preceding mobilization of the cells to areas of inflammation [49]. He suggested that the peritoneal cavity might act as a “culture chamber” where macrophages would arise before their mobilization to distant inflammatory sites [48]. Therefore, inactivation of peritoneal macrophages by DHMEQ may contribute to suppress macrophage activity in the peripheral sites.

**Figure 9:**
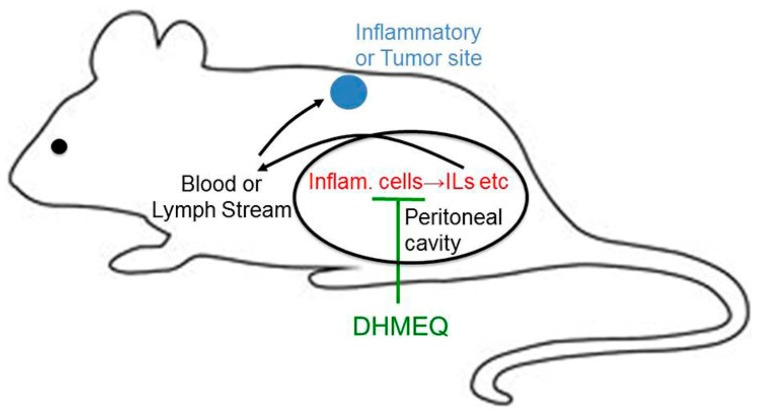
Peritoneal NF-κB may contribute to the progression of peripheral inflammation and cancer.

It may be a possible theory that the regulation of NF-κB in the cells of peritoneal cavity would influence many peripheral inflammation and cancer (Figure 9). IP administration of immuno-suppressing agent including DHMEQ may be useful for the treatment of cancer metastasis and other difficult diseases in the future.

## 7. Conclusions and Future Prospective

**Figure 10:**
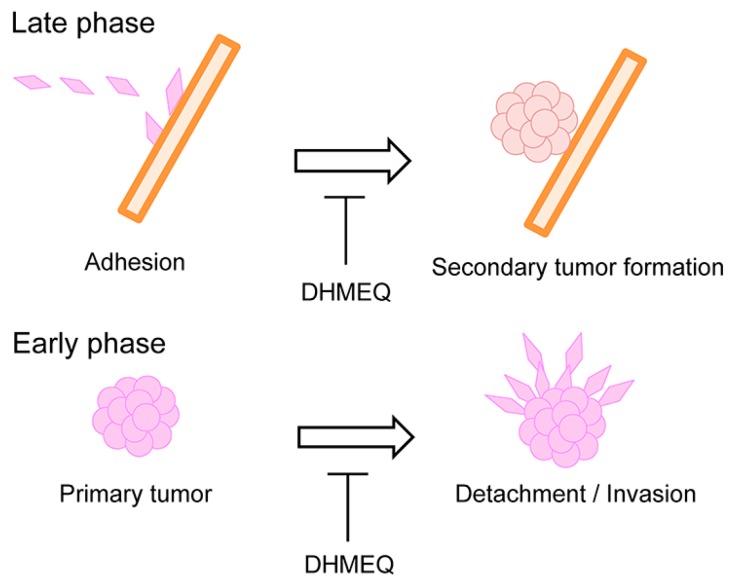
DHMEQ would inhibit both late and early phases of metastasis.

The late phase of metastasis is commonly studied by injection of cancer cells into the transporting blood vessels. DHMEQ inhibited traditional in vivo metastasis models that are for the late phase of metastasis. On the other hand, we first employed 3D culture of human breast cancer MDA-MB-231 cells as a model of the early phase of metastasis. Meanwhile DHMEQ derived from microbial bioactive metabolite epoxyquinomicin C is a specific inhibitor of NF-κB. DHMEQ inhibited the models of both the late and early phase metastasis (Figure 10). Since DHMEQ is nontoxic in animals, it may become a useful anti-metastatic agent.

## References

[1] Onoda T., Iinuma H., Sasaki Y., Hamada M., Isshiki K., Naganawa H., Takeuchi T., Tatsuta K., Umezawa K. (1989). Isolation of a novel tyrosine kinase inhibitor, lavendustin A, from *Streptomyces griseolavendus*. J. Nat. Prod..

[2] Imoto M., Kakeya H., Sawa T., Hayashi C., Hamada M., Takeuchi T., Umezawa K. (1993). Dephostasin, a novel protein tyrosine phosphatase inhibitor produced by *Streptomyce*s. I. Taxonomy, isolation, and characterization. J. Antibiot..

[3] Ohse T., Ohba S., Yamamoto T., Koyano T., Umezawa K. (1996). Cyclopentabenzofuran lignan protein synthesis inhibitors from *Aglaia odorata*. J. Nat. Prod..

[4] Fukami A., Ikeda Y., Kondo S., Naganawa H., Takeuchi T., Furuya S., Hirabayashi Y., Shimoike K., Hosaka S., Watanabe Y. (1997). Akaterpin, a novel bioactive triterpene from the marine sponge *Callyspongia sp.*. Terrahedron Lett..

[5] Ohno O., Ikeda Y., Sawa R., Igarashi M., Kinoshita N., Suzuki Y., Miyake K., Umezawa K. (2004). Isolation of heptadepsin, a novel bacterial cyclic depsipeptide that inhibits lipopolysaccharide activity. Chem. Biol..

[6] Arai Y., Iinuma H., Ikeda Y., Igarashi M., Hatano K., Kinoshita N., Ukaji T., Simizu S., Umezawa K. (2013). Migracins, A and B, new inhibitors of cancer cell migration, produced by *Streptomyces* sp.. J. Antibiot..

[7] Umezawa K., Kojima I., Simizu S., Lin Y.Z., Fukatsu H., Koide N., Nakade Y., Yoneda M. (2017). Therapeutic a activity of plant-derived alkaloid conophylline on metabolic syndrome and neurodegenerative disease models. Hum. Cell.

[8] Matsumoto N., Ariga A., To-e S., Nakamura H., Agata N., Hirano S., Inoue J., Umezawa K. (2000). Synthesis of NF-κB activation inhibitors derived from epoxyquinomicin C. Bioorg. Med. Chem. Lett..

[9] Erkel G., Anke T., Sterner O. (1996). Inhibition of NF-κB activation by panepoxydone. Biochem. Biophys. Res. Commun..

[10] Gehrt A., Erkel G., Anke T., Sterner O.A. (1998). Cycloepoxydon: 1-hydroxy-2-hydroxymethyl-3-pent-1-enylbenzene1-hydroxy-2-hydroxymethyl-3-pent-1,3-dienylbenzene, new inhibitors of eukaryotic signal transduction. J. Antibiot..

[11] Suzuki Y., Sugiyama C., Ohno O., Umezawa K. (2004). Preparation and biological activities of optically active dehydroxymethylepoxyquinomicin, a novel NF-κB inhibitor. Tetrahedron.

[12] Hamada M., Niitsu Y., Hiraoka C., Kozawa I., Higashi T., Shoji M., Umezawa K., Sugai T. (2010). Chemoenzymatic synthesis of (2S,3S,4S)-form, the physiologically active stereoisomer of dehydroxymethylepoxyquinomicin (DHMEQ), a potent inhibitor on NF-κB. Tetrahedron.

[13] Ariga A., Namekawa J., Matsumoto N., Inoue J., Umezawa K. (2002). Inhibition of TNF-α-induced nuclear translocation and activation of NF-κB by dehydroxymethyl-epoxyquinomicin. J. Biol. Chem..

[14] Yamamoto M., Horie R., Takeiri M., Kozawa I., Umezawa K. (2008). Inactivation of nuclear factor kappa B components by covalent binding of (−)-dehydroxymethylepoxyquinomicin to specific cysteine residues. J. Med. Chem..

[15] Horie K., Ma J., Umezawa K. (2015). Inhibition of canonical NF-κB nuclear localization by (−)-DHMEQ via impairment of DNA binding. Oncol. Res..

[16] Kozawa I., Kato K., Teruya T., Suenaga K., Umezawa K. (2009). Unusual intramolecular N→O acyl group migration occurring during conjugation of (−)-DHMEQ with cysteine. Bioorg. Med. Chem. Lett..

[17] Shimada C., Ninomiya Y., Suzuki E., Umezawa K. (2010). Efficient cellular uptake of the novel NF-κB inhibitor (−)-DHMEQ and irreversible inhibition of NF-κB in neoplastic cells. Oncol. Res..

[18] Takeiri M., Horie K., Takahashi D., Watanabe M., Horie R., Simizu S., Umezawa K. (2012). Involvement of DNA binding domain in the cellular stability and importin affinity of NF-κB component RelB. Org. Biomol. Chem..

[19] Hamasaka A., Yoshioka N., Abe R., Kishino S., Umezawa K., Ozaki M., Todo S., Shimizu H. (2010). Topical application of DHMEQ improves allergic inflammation via NF-κB inhibition. J. Allergy Clin. Immunol..

[20] Noma N., Asagiri M., Takeiri M., Ohmae S., Takemoto K., Iwaisako K., Simizu S., Umezawa K. (2015). Inhibition of MMP-2-mediated mast cell invasion by NF-κB inhibitor DHMEQ in mast cells. Int. Achieves Allergy Immunol..

[21] Jiang X., Wei B., Lan Y., Dai C., Gu Y., Ma J., Liu X., Umezawa K., Zhang Y. (2017). External application of NF-κB inhibitor DHMEQ suppresses development of atopic dermatitis-like lesions induced with DNCB/OX in BALB/c mice. Immunopharmacol. Immunotoxicol..

[22] Kobayashi K., Umezawa K., Yasui M. (2011). Apoptosis in mouse amniotic epithelium is induced by activated macrophages through the TNF receptor type 1/TNF pathway. Biol. Reprod..

[23] Yamanouchi S., Adachi Y., Shimo T., Umezawa K., Okigaki M., Tsuji S., Li M., Takaya J., Kuge T., Ikehara S., Kaneko K. (2015). A nuclear factor-κB inhibitor, dehydroxymethylepoxyquinomicin, ameliorates GVHD in allogeneic bone marrow transplantation. Immunobiology.

[24] El-Salhy M., Umezawa K. (2016). Effects of AP-1 and NF-kappa B inhibitors on colonic endocrine cells in rats with TNBS-induced colitis. Mol. Med. Rep..

[25] El-Salhy M., Umezawa K. (2016). Anti-inflammatory effects of novel AP-1 and NF-κB inhibitors in dextran-sulfate-sodium-induced colitis in rats. Int. J. Mol. Med..

[26] Nishio H., Yaguchi T., Sugiyama J., Sumimoto H., Umezawa K., Iwata T., Susumu N., Fujii T., Kawamura N., Kobayashi A. (2014). Immunosuppression through constitutively activated NF-κB signaling in human ovarian cancer and its reversal by a NF-κB inhibitor. Br. J. Cancer.

[27] Seubwai W., Kraiklang R., Vaeteewoottacharn K., Umezawa K., Okada S., Wongkham S. (2014). Aberrant expression of NF-κB in liver fluke associated cholangiocarcinoma: Implications for targeted therapy. PLoS ONE.

[28] Ito Y., Kikuchi E., Tanaka N., Kosaki T., Suzuki E., Mizuno R., Shinojima T., Miyajima A., Umezawa K., Oya M. (2015). Down-regulation of NF kappa B activation is an effective therapeutic modality in acquired platinum-resistant bladder cancer. BMC Cancer.

[29] Miyake A., Dewan A., Ishida T., Watanabe M., Honda M., Sata T., Yamamoto N., Umezawa K., Watanabe T., Horie R. (2008). Induction of apoptosis in Epstein-Barr virus-infected B-lymphocytes by the NF-κB inhibitor DHMEQ. Microbes Infect..

[30] Watanabe M., Ohsugi T., Shoda M., Ishida T., Aizawa S., Maruyama-Nagai M., Utsunomiya A., Koga S., Yamada Y., Kamihira S. (2005). Dual targeting of transformed and untransformed HTLV-1-infected T-cells by DHMEQ, a potent and selective inhibitor of NF-κB, as a strategy for chemoprevention and therapy of adult T cell leukemia. Blood.

[31] Saito D., Sawamura M., Umezawa K., Kanai Y., Furihata C., Matsushima T., Sugimura T. (1980). Inhibition of experimental blood-borne lung metastasis by protease inhibitors. Cancer Res..

[32] Suzuki K., Aiura K., Matsuda S., Itano O., Takeuchi O., Umezawa K., Kitagawa Y. (2013). Combined effect of dehydroxymethylepoxyquinomicin and gemcitabine in a mouse model of liver metastasis of pancreatic cancer. Clin. Exp. Metastasis.

[33] Sato M., Nakanishi K., Haga S., Fujiyoshi M., Baba M., Mino K.Y., Niwa H., Yokoo H., Umezawa K., Ohmiya Y. (2014). Anoikis induction and inhibition of peritoneal metastasis of pancreatic cancer cells by a nuclear factor-kappa B inhibitor, (−)-DHMEQ. Oncol. Res..

[34] Ukaji T., Lin Y.Z., Okada S., Umezawa K. (2017). Inhibition of MMP-2-mediated cellular invasion by NF-κB inhibitor DHMEQ in 3D culture of breast carcinoma MDA-MB-231 cells: A model for early phase of metastasis. Biochem. Biophys. Res. Commun..

[35] Wakamatsu K., Nanki T., Miyasaka N., Umezawa K., Kubota T. (2003). Effect of a small molecule inhibitor of nuclear factor-κB nuclear translocation in a murine model of arthritis and cultured human synovial cells. Arthritis Res. Ther..

[36] Ohsugi T., Horie R., Kumasaka T., Ishida A., Ishida T., Yamaguchi K., Watanabe T., Umezawa K., Urano T. (2005). In vivo antitumor activity of the NF-κB inhibitor dehydroxymethyl-epoxyquinomicin in a mouse model of adult T-cell leukemia. Carcinogenesis.

[37] Umezawa K. (2011). Possible role of peritoneal NF-κB in peripheral inflammation and cancer: Lessons from the inhibitor DHMEQ. Biomed. Pharmacother..

[38] Umezawa K. (2013). Peritoneal NF-κB as a Possible molecular target for suppression of various cancers and inflammation. Forum Immunopathol. Dis. Ther..

[39] Watanabe E., Mochizuki N., Ajima H., Ohno K., Shiino M., Umezawa K., Fukai M., Ozaki M., Furukawa H., Todo S. (2008). A simple and reliable method for determining plasma concentration of dehydroxymethylepoxyquinomicin by high performance liquid chromatography with mass spectrometry. J. Chromatogr. B.

[40] Saitoh T., Suzuki E., Takasugi A., Obata R., Ishikawa Y., Umezawa K., Nishiyama S. (2009). Efficient synthesis of (±)-parasitenone, a novel inhibitor of NF-κB. Bioorg. Med. Chem. Lett..

[41] Park B.K., Zhang H., Zeng Q., Dai J., Keller E.T., Giordano T., Gu K., Shah V., Pei L., Zarbo R.J. (2007). NF-κB in breast cancer cells promotes osteolytic bone metastasis by inducing osteoclastogenesis via GM-CSF. Nat. Med..

[42] Zins K., Abraham D., Sioud M., Aharinejad S. (2007). Colon cancer cell–derived tumor necrosis factor-α mediates the tumor growth–promoting response in macrophages by up-regulating the colony-stimulating factor-1 pathway. Cancer Res..

[43] Ukaji T., Umezawa K. (2014). Novel approaches to target NF-κB and other signaling pathways in cancer stem cells. Adv. Biol. Regul..

[44] Nagai N., Oike Y., Izumi-Nagai K., Koto T., Satofuka S., Ozawa Y., Yamashiro K., Inoue M., Tsubota K., Umezawa K. (2007). Suppression of diabetes-induced retinal inflammation by blocking angiotensin II type 1 receptor or its downstream NF-κB pathway. Investig. Ophthalmol. Vis. Sci..

[45] Fukushima T., Kawaguchi M., Yorita K., Tanaka H., Umezawa K., Kataoka H. (2012). Antitumor effect of dehydroxymethylepoxyquinomicin (DHMEQ), a small molecule inhibitor of nuclear factor-κB, on glioblastoma. Neuro-Oncology.

[46] Kuroda K., Horiguchi Y., Nakashima J., Kikuchi E., Kanao K., Miyajima A., Ohigashi T., Umezawa K., Murai M. (2005). Prevention of cancer cachexia by a novel nuclear factor κB inhibitor in prostate cancer. Clin. Cancer Res..

[47] Suzuki E., Sugiyama C., Umezawa K. (2009). Inhibition of inflammatory mediator secretion by (−)-DHMEQ in mouse bone marrow-derived macrophages. Biomed. Pharmacother..

[48] Sosinnska P., Macckowiak B., Staniszewski R., Umezawa K., Breborowicz A. (2016). Inhibition of NF-κB with dehydroxyepoxiquinomicin modifies function of human peritoneal mesothelial cells. Am. J. Transl. Res..

[49] Vernon-Roberts B. (1969). Lymphocyte to macrophage transformation in the peritoneal cavity preceding the mobilization of peritoneal macrophages to inflamed areas. Nature.

